# Virtual Reality Interventions for Needle-Related Procedural Pain, Fear and Anxiety—A Systematic Review and Meta-Analysis

**DOI:** 10.3390/jcm10153248

**Published:** 2021-07-23

**Authors:** Oliver Czech, Adam Wrzeciono, Anna Rutkowska, Agnieszka Guzik, Paweł Kiper, Sebastian Rutkowski

**Affiliations:** 1 Descartes’ Error Student Research Association, Faculty of Physical Education and Physiotherapy, Opole University of Technology, 45-758 Opole, Poland; oliver.czech@student.po.edu.pl (O.C.); adam.wrzeciono@student.po.edu.pl (A.W.); 2 Department of Physical Education and Physiotherapy, Opole University of Technology, 45-758 Opole, Poland; a.rutkowska@po.edu.pl; 3 Department of Physiotherapy, Institute of Health Sciences, College of Medical Sciences, University of Rzeszów, 35-310 Rzeszów, Poland; agnieszkadepa2@wp.pl; 4 Physical Medicine and Rehabilitation Unit, Azienda ULSS 3 Serenissima, 30126 Venice, Italy; pawelkiper@hotmail.com

**Keywords:** virtual reality, VR, needle, invasive procedures, pain, anxiety, fear

## Abstract

Needle-related procedures are often a source of pain, anxiety and fear in young patients. This systematic review aimed to investigate the effectiveness of virtual reality (VR) on reducing pain, fear and anxiety in pediatric patients undergoing needle-related procedures. Pain, anxiety, fear, changes in blood pressure and heart rate as well as satisfaction were evaluated as outcomes during needle-related procedures in VR compared with standard care conditions. A meta-analysis was performed, taking into account online databases. Two authors independently conducted literature searches in December 2020. The last search was conducted in March 2021 from a total of 106 records, 7 met our inclusion criteria. One study was excluded from the meta-analysis due to insufficient data. VR was applied as a distractor during venous access. Statistically significant benefits of using VR were shown in children’s pain scores, where VR significantly decreased symptoms (*n* = 3204 patients, MD = −2.85; 95% CI −3.57, −2.14, for the Wong–Baker Faces Pain Rating Scale and *n* = 2240 patients, MD = −0.19; 95% CI −0.58, 0.20, for the Faces Pain Scale—Revised). The analysis of fear, anxiety and satisfaction scores revealed no significant differences between the conditions, as the studies were too heterogeneous to be pooled. Distraction using virtual reality may be an effective intervention for reducing pain in children undergoing needle-related medical procedures. However, further research in the implementation of VR as a distractor for children and adolescents is required, due to the limited research into this field.

## 1. Introduction

Invasive procedures are an integral part of the diagnosis and treatment program in various diseases. In many such interventions, needle use is required. The needle-related procedures are frequently accompanied by anxiety or fear. It has been shown that psychological factors can affect in various ways the sensation of pain [1,2,3]. The literature suggests that stress, anxiety and fear may not only increase pain but can also induce it [4,5]. Especially for young patients, invasive interventions are very stressful. Children often report concerns even before the procedure. Such anticipatory fear may also cause an increase in pain resulting in emotional distress [6].

Frequent stress and anxiety associated with needle-related procedures may lead to needle phobia [7]. Already at the end of the 20th century, it was noticed that an individual approach should be taken to treating pain associated with a procedure using behavioral and pharmacological interventions [8]. Because pain perception has both sensory (pain stimulus) and affective (depression, fear, anger etc.) components, it is believed that distraction methods may be an effective tool in reducing patients’ pain during various invasive procedures.

Research in recent years has focused on the development of effective distraction methods for needle-related procedures in young patients. Many distraction techniques have been tested. Music, massage, breathing exercises, and behavioral therapy were found to be possible effective distracting methods [9,10,11]. Various studies show that distraction reduces the feeling of pain and leads to a reduction in stress symptoms during painful procedures [12,13,14]. Distraction involves a process by which the patient’s senses become disconnected from the nociceptive stimulus. An effective distractor should be immersive, by stimulating as many senses as possible, and highly engaging. Research shows that combining visual and audio distraction stimuli is more effective than using visual stimuli alone [15]. An example of such a distractor is virtual reality (VR).

VR has been used to manage the pain and anxiety associated with medical procedures [16]. The literature identifies four types of VR: immersive virtual reality, non-immersive (desktop) virtual reality, augmented virtual reality and mixed virtual reality (a combination of real objects and environments with virtual people or places) [17]. The value of the application of immersive VR in clinical settings is that immersive VR environments can enable researchers or clinicians to modify multimodal input stimuli to make patients feel “present” in the projected environment [18,19]. VR is a combination of specialized hardware and software. With modern VR technology combined with imaging examinations, surgeons can project internal organs in real-time during the operation, which minimizes the risk of damage, even in the most complex conditions. This type of technology can be interesting and engaging for children [20]. An immersive VR allows medical professionals to efficiently affect the feeling of pain in patients undergoing painful procedures, by completely distracting attention from the stimulus. Research shows the beneficial impact of using VR as a distraction during dental care in children [21], burn wound care, oncological treatment, venous access and dental interventions [22]. In addition, the side effects of short-term VR applications are usually harmless and rare. The risk of dizziness, headache, nausea and eye strain increases if the patient is in VR for more than 20 min [15]. VR interventions have been used by psychologists, pediatricians, neurologists and physiotherapists [23,24]. According to research, in the treatment of depression, it may be effective to transfer the patient to a virtual world [25,26]. Thanks to immersion, the affected patient can, at least for a moment, forget about the problems of everyday life and move to a place filled with peace and positive energy. Non-immersive forms of VR have proven beneficial in rehabilitation. Systems such as Neuroforma, Xbox Kinect, or Nintendo Wii help patients regain and maintain, physical fitness [27], balance [28], or motor function [29]. However, to date, the effectiveness of VR interventions during hospital needle-related medical procedures has not been confirmed by a meta-analysis. Therefore, this systematic review aimed to analyze and synthesize the evidence on the effectiveness of virtual reality interventions in the prevention of pain, fear and anxiety during needle-related procedures. The included studies were based on the irrigation of drips and cannulation and blood draw. All treatments cause pain of comparable intensity, because of the use of similar-sized needles.

## 2. Methods

The study was designed as a systematic review with meta-analysis and followed the Preferred Reporting Items for Systematic Reviews and Meta-Analyses (PRISMA) guidelines for reporting systematic reviews [30]. The protocol was registered a priori in the PROSPERO database with the registration number: CRD42021216447.

### 2.1. Electronic Searches

Two authors independently conducted literature searches in December 2020, using the following electronic databases: PubMed (National Library of Medicine, 8600 Rockville Pike, Bethesda, MD 20894, United States), Cochrane Library (The Cochrane Collaboration, St Albans House, 57–59 Haymarket, London, UK), Web of Science (Clarivate, 1500 Spring Garden, Philadelphia, PA 19130, USA), Scopus (Elsevier, Radarweg 29, 1043 NX Amsterdam, The Netherlands) and Embase (Elsevier, Radarweg 29, 1043 NX Amsterdam, The Netherlands). The last search was made in March 2021. Due to the limited amount of research available, no specific publication dates were determined. The following medical subject headings (MeSH) search terms were defined: “Virtual Reality Exposure Therapy”, “Smart Glasses”, “Virtual Reality”, “Acute Pain”, “Pain”, “Fear”, “Anxiety”, “Heart Rate” and “Blood Pressure”. A full description of the search strategy is presented in Supplementary Materials (File S1). 

### 2.2. Study Selection

We included: (1) studies designed as randomized controlled trials (RCTs), (2) children, without gender or diagnosis restrictions, including patients that underwent needle-related medical procedures (e.g., injections, intravenous infusions, lumbar punctures, etc.), (3) interventions defined as immersive, non-immersive or mixed-reality VR scenarios that allow the patient to be distracted during the medical intervention and (4) with measured outcomes associated with pain level, fear or anxiety level, changes in blood pressure or heart rate. The present report includes studies in English, Polish and Italian. Grey literature was also searched on Google Scholar databases (Google, 1600 Amphitheatre Parkway, Mountain View, CA, USA). Two reviewers independently screened the studies’ abstracts using an inclusion/exclusion criteria template, with the intervention of a third researcher in case of disagreement. In the next step, the full texts were screened and assessed for the methodological quality (risk of bias assessment) with the same procedures.

### 2.3. Outcomes

The primary outcomes were patients’ feelings of fear, anxiety or pain during hospital needle-related procedures. The outcomes were analyzed in experimental and control conditions. Virtual reality was used in the experimental groups, compared to other (traditional) interventions that help to distract attention or in comparison to no intervention. Secondary outcomes were related to changes in blood pressure or heart rate during VR scenarios as well as the satisfaction level after the medical procedure.

### 2.4. Data Extraction and Management

All the relevant data were entered into the data extraction form, i.e., authors, year of publication, study design, participants’ characteristics, attrition from intervention, co-interventions, number of participants, details of intervention procedures, outcome measures and when they were administered.

### 2.5. Assessment of Risk of Bias in Included Studies

The Review Manager software (RevMan version 5.4; The Nordic Cochrane Centre, The Cochrane Collaboration, St Albans House, 57–59 Haymarket, London, the United Kingdom) and the Cochrane Risk of Bias Tool [31] were used for a methodological quality assessment for risk of bias of the included studies. The following domains were evaluated: (1) selection bias: sequence generation, allocation concealment; (2) detection bias: blinding of outcome assessment; (3) attrition bias: incomplete outcome data; and (4) reporting bias: selective reporting. In the case of a low possibility of bias, the studies were categorized as “low risk”, in the case of a high possibility of bias—“high risk” and if the occurrence of risk of bias could not be indicated—“unclear risk”. An in-detail summary of the risk of bias assessment is included in Supplementary Materials (File S2). As participant blinding in most cases of virtual reality intervention is not possible, it was decided to omit the domain of assessment of participant blinding. Judging a result to be at a particular level of risk of bias for an individual domain implies that the result has an overall risk of bias at least severe. Therefore, a judgement of “High” risk of bias within any domain should have similar implications for the result, irrespective of which domain is being assessed.

### 2.6. Data Synthesis and Statistical Analysis

The statistical analysis and meta-analysis were conducted using RevMan 5.4.1 Standardized Mean Difference (SMD) for outcomes measured with different scales and Mean Difference (MD) for homogeneous outcome measures. Statistical heterogeneity was assessed with the I^2^ statistic, establishing the cut-off value at 50% and considering intervention and outcome measures. The confidence interval (CI) for continuous outcomes was identified at 95%. A meta-analysis was conducted based on a random-effects model or fixed model with 95% CI. As in some of the included studies the parent-reported as well as the patient-reported pain, fear, anxiety and satisfaction were assessed, it was decided to analyze and include the parent-reported measured outcomes. It was also decided to calculate the effect size (ES) of the included studies. In relation to the study design, we used two: Cohen’s *d*; considered as small (0.0–0.2), medium (>0.2 and <0.5) or large (>0.5) [32], or Morris *d*; classified as small (0.1–0.3), intermediate (0.3–0.5) and large (≥0.5) [33].

## 3. Results

**Figure 1:**
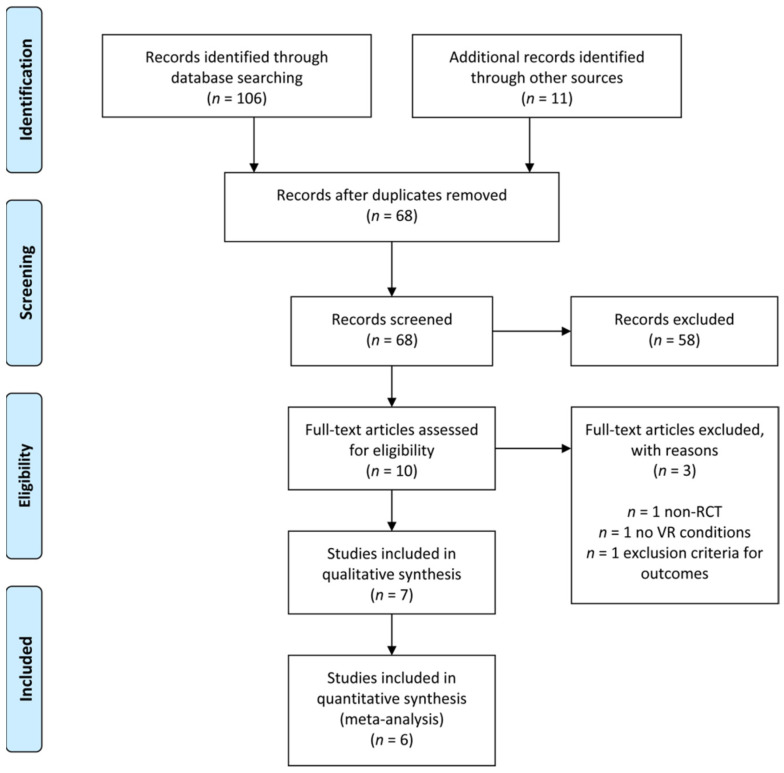
Flow diagram of the studies.

The electronic search identified 106 results overall, and 11 studies were added from the grey literature search. After removing 49 duplicates, 68 abstracts were included for screening. 58 records were excluded due to their unrelated topic and 10 full-text articles were assessed for eligibility. Finally, 7 studies met the inclusion criteria and underwent a qualitative analysis. Three full-text articles were excluded after eligibility assessment, due to different reasons: *n* = 1—non RCT, *n* = 1 no VR environment and *n* = 1 exclusion criteria for outcomes. One further study was excluded due to insufficient data, leaving six studies that were included in the meta-analysis. The PRISMA flowchart presents the review process (Figure 1).

### 3.1. Included Studies

#### 3.1.1. Characteristic of Included Studies

All of the included publications were randomized clinical trials focused on the use of VR as a distractor in needle-related medical procedures. In all studies, participants represented both sexes and were under 18 years of age (range 5–18). The procedure included basic medical activities related to blood sampling or intravenous placement. Studies included participants from: emergency departments [34,35], departments of radiology [34,36,37], oncology units [34,38,39] and blood drawing units [34,40]. The overall number of participants within the trials was 554. Most of the studies also investigated the feelings of the participants’ parents, with an overall number of 304 participants [35,36,37,38,40].

The study by Caruso et al. included a comparison of pain perception between a VR group and a standard care group. Secondary outcomes consisted of assessment of fear, satisfaction and procedural compliance. They used a VR system, Samsung Gear Oculus, with one of three VR experiences: Ocean Rift, Pebbles the Penguin or Space Pups, displayed during the procedure. The authors noticed non-significant differences in post-procedure pain (*p* = 0.62), fear (*p* = 0.58) and compliance (*p* = 0.69) [34].

Dumoulin et al. instead of an interactive movie used an immersive fly shooting game to compare with a standard care group and a non-immersive TV distractor. The primary outcomes measured were pain and anxiety. Patient satisfaction and negative side effects were also studied. The comparison of the three conditions showed a significant improvement in satisfaction in the children’s ratings in the VR group (*p* < 0.01) [35].

In the study by Gerçeker et al. (2020) the Samsung Gear Oculus headset was used. Virtual reality experiences, like Ocean Rift, Rillix VR and “In the eyes of animal”, were compared to standard care procedures. The examined parameters included scores for pain, anxiety and fear. A statistically significant difference was found between groups according to the self-, parent-, researcher- and nurse-reported pain scores (*p* < 0.05). The VR-Rollercoaster group and the VR-Ocean Rift group had no statistically significant superiority over each other (*p* < 0.05). A statistically significant difference was found between groups according to the fear and anxiety scores after a blood draw (*p* < 0.05) [36].

Gerçeker et al. (2021) in their other work also used the Samsung Gear Oculus headset. In this study, the VR group was divided into two experimental groups: Ocean Rift and Rillix VR, compared to the standard care. The primary outcomes remained unchanged compared to the surveys of the previous year. This study found a statistically significant difference between groups in pain scores (*p* < 0.001). A statistically significant improvement was found in the self-and parent-reported CFS fear and CAS-D anxiety scores after the procedure (*p* < 0.001) [40].

Gold et al. evaluated the efficacy and suitability of VR as a pain distraction for pediatric intravenous placement. A VR group playing “Street Luge” was compared to a standard care group. Primary outcomes were anticipatory anxiety, affective pain, pain intensity, measures of past procedural pain and satisfaction. No significant differences were reported between the treatment groups on any measures of affective pain and anticipatory anxiety for children and their parents [37].

A study by Semerci et al. compared a rollercoaster VRET distractor to a standard care group. The only tested outcome was the pain score. There was a significant difference between the control and VR group (*p* = 0.001). The mean pain score of the children in the control group was significantly higher than that of the VR group [38].

In all studies, pain was assessed using the VAS or FACES scales. For measuring fear score the Children’s Fear Scale (CFS) was used. For measuring the anxiety level the Childhood Anxiety Sensitivity Index and The Children’s Anxiety Meter-State were used.

The results of a study by Sander Wint et al. have not been included in the meta-analysis, due to lack of data. The patients in these studies underwent a lumbar puncture. The comparison concerned a standard care group and a group watching VR movies. The measured outcomes were pain score, sedation level and experience during the lumbar puncture. Although no statistically significant difference was found (*p* = 0.77) on the VAS pain scores between the control and experimental groups, those in the VR group reported a trend toward lower pain scores [39].

The characteristics of the included studies are presented in detail in Supplementary Materials (File S3).

#### 3.1.2. Effect Size of Included Studies

Four of the included studies noted a large effect size [36,37,38,40], whereas studies by Caruso et al. [34] and Dumoulin et al. [41] noted a small effect size of VR interventions. The data of the study by Wint et al. [39] were insufficient to assess the effect size.

### 3.2. Excluded Studies

Three studies were excluded after a screening of full texts. One study was considered ineligible as it was non-RCT [41], while one study did not use VR conditions [42]. Finally, one study was excluded as the primary outcomes did not fit the review assumptions. The measured outcomes were safety concerns, effectiveness, usability and engagement of VR during intravenous procedures [43].

### 3.3. Risk of Bias of the Included Studies

Random sequence generation (selection bias): Five studies had a low risk of bias [34,35,36,38,40]. The authors described in detail a random component of the sequence-generation process. Two studies were assessed as having an unclear risk of bias, as no information about the randomization process was provided [37,39]. 

Allocation concealment (selection bias): Three studies were judged at low risk of bias, as the allocation methods used were appropriate [34,36,38]. One study had a high risk of bias because allocation was based on gender and age group [40]. Three studies were assessed with an unclear risk of bias as they contained no information about allocation concealment procedures [35,37,39].

Blinding of outcome assessment (detection bias): In one study the data analyst was blinded, so the risk of bias was judged as low [40]. One study was assessed with a high risk, as the study was not blinded [34]. Five studies were judged with an unclear risk, due to lack of information about blinding of assessors [34,35,36,37,39]. 

Incomplete outcome data (attrition bias): Six studies were assessed with a low risk of bias because no missing data were found, or the purpose of participants’ exclusion was properly argued [35,36,37,38,39,40]. Only one study had a high risk of bias because the number of drop-outs due to missing primary outcome data was high [34]. 

**Figure 2:**
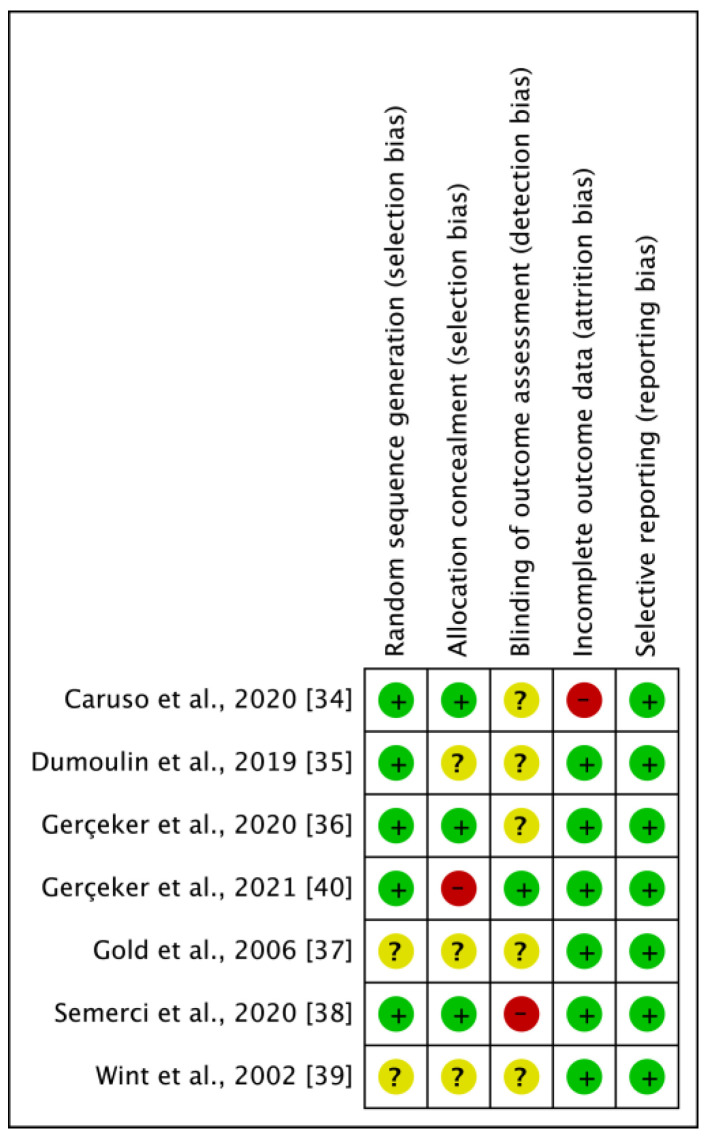
Risk of bias summary. + (green): low risk; ? (yellow): unclear risk; − (red): high risk.

Selective reporting (reporting bias): All of the seven studies were judged with a low risk of bias. Some of the studies’ protocols were available, and some were not, but in either case the published reports include all expected outcomes. Figure 2 shows the risk of bias in the included studies.

The overall risk of bias assessment results indicate that three publications have a high risk of bias [31,35,37]. The remaining four studies are considered to be of average risk of bias. Such a result obliges to be cautious in drawing conclusions. 

### 3.4. Effects of Intervention

#### 3.4.1. Comparison of VR Treatment and Standard Care. Outcome: Pain

**Figure 3:**
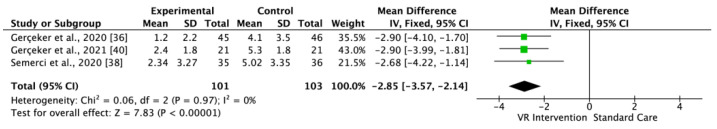
Comparison of VR treatment and standard care, pain (WBS). SD: standard deviation; 95% CI: 95% confidence interval; green square: mean difference; black rhombus: pooled effect.

Three studies, with an overall number of 204 patients, were analyzed for pain score after a needle-related procedure. As the outcome measures in the included studies were performed using the Wong–Baker Faces Pain Rating Scale (WBS), the analysis was performed using MD with a fixed effect model. The meta-analysis showed a significant difference between the two treatment conditions (MD = −2.85; 95% CI −3.57, −2.14; I^2^ = 0%) (Figure 3). 

**Figure 4:**
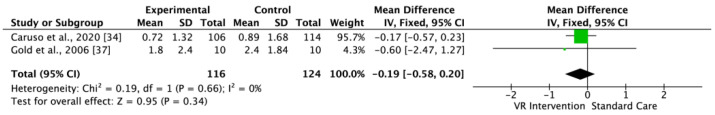
Comparison of VR treatment and standard care, pain (FPS-R). SD: standard deviation; 95% CI: 95% confidence interval; green square: mean difference; black rhombus: pooled effect.

Two studies, with 240 patients overall, were analyzed for pain score after a needle-related procedure. As the outcome measures in the included studies were performed using the Faces Pain Scale–Revised (FPS-R), the analysis was performed using MD with a fixed effect model. No significant difference was found between VR Treatment and standard care for pain score (MD = −0.19; 95% CI −0.58, 0.20; I^2^ = 0%) (Figure 4).

#### 3.4.2. Comparison of VR Treatment and Standard Care. Outcome: Fear

**Figure 5:**
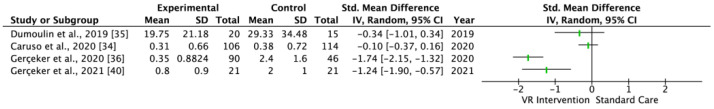
Comparison of VR treatment and standard care, fear. SD: standard deviation; 95% CI: 95% confidence interval. Without pooling due to heterogeneity (I^2^ = 94%).

Four studies, with 433 patients overall, were analyzed for fear symptoms after a needle-related procedure. As in the included studies the fear score was assessed using different scales, the analysis was performed using SMD with random model effect. As presented in Figure 5, these studies were too heterogeneous to be pooled (I^2^ = 94%). 

#### 3.4.3. Comparison of VR Treatment and Standard Care. Outcome: Anxiety

**Figure 6:**
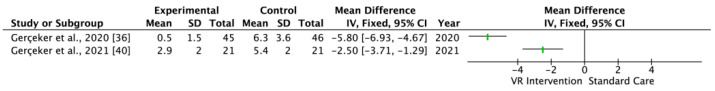
Comparison of VR treatment and standard care, anxiety (CAM). SD: standard deviation; 95% CI: 95% confidence interval. Without pooling due to heterogeneity (I^2^ = 93%).

A total of two studies, with an overall number of 133 participants, were analyzed for anxiety scores after a needle-related procedure. The analysis was performed using MD with a fixed effect model, as the outcome measures in the included studies were conducted using The Children’s Anxiety Meter (CAM). These studies were too heterogeneous to be pooled (I^2^ = 93%) as presented in Figure 6.

#### 3.4.4. Comparison of VR Treatment and Standard Care. Outcome: Satisfaction

**Figure 7:**
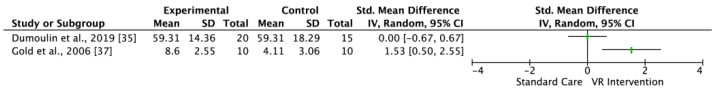
Comparison of VR treatment and standard care, satisfaction. SD: standard deviation; 95% CI: 95% confidence interval. Without pooling due to heterogeneity (I^2^ = 83%).

Two studies, with 55 patients overall, were analyzed for satisfaction level after a needle-related procedure. As the outcome measures in the included studies were different, the analysis was performed using SMD with a random effect model. As presented in Figure 7, these studies were too heterogeneous to be pooled (I^2^ = 83%).

## 4. Discussion

This systematic review and meta-analysis aimed to assess the effectiveness of VR interventions as a distractor in needle-related medical procedures carried out on children, compared to standard care methods. Statistically significant benefits of using VR were shown in children’s pain scores, where VR significantly decreased symptoms. The analysis of fear, anxiety and satisfaction scores revealed no significant differences between the conditions. The results suggest that VR has the potential to become an important tool in decreasing pain in young patients undergoing needle-related procedures in a variety of medical settings. However, the included studies involved various age groups and medical settings, and it is not possible to determine for which age group and during which needle-related procedure VR may be the most effective as a distraction tool. Therefore there is no possibility to clearly define the clinical importance of VR as a distractor. Further research, with a similar study design, could contribute to a more precise evaluation of VR as a distraction tool. The current access to the results and the number of studies performed do not allow for an unambiguous determination of clinical importance. Estimates of the minimum clinically important difference (MCID) depends on the type of pain, the starting pain level and other factors, but they tend to range from 10% to 25%—in other words, a change of one face on the FPS-R would be the smallest meaningful change. However, this information does not affect the results, because the meta-analysis showed no statistically significant change in FPS-R measures. A meta-analysis by Eijlers et al. found a significant decrease in patient-reported pain and anxiety in pediatric patients undergoing a range of medical procedures with VR used as a distractor. The large effect size could lead to the drawing of specific conclusions: as a method for reducing pain and anxiety in pediatric patients undergoing medical procedures, VR is an effective distraction intervention [22]. The effectiveness of VR as a distraction method is also confirmed by the study of Valverde et al. examining pain and anxiety sensations in children being distracted by virtual reality during dental procedures. The results show a significant reduction of pain and anxiety [44]. It suggests the usefulness of VR during medical procedures in children. The value of this type of research is also confirmed by the WHO, which considers the fear of dental procedures to be a serious problem in 15–20% of the population [45].

Generalizing the results in terms of the usefulness of virtual reality in reducing symptoms of fear and anxiety, as well as improving the satisfaction resulting from therapy may prove to be impossible, due to the high heterogeneity of the results. This heterogeneity could have been caused by differences between the treatments that were performed. Some treatments generate more anxiety symptoms, others fewer. The pain involved in the treatments is also different. 

The beneficial effect of virtual reality in reducing pain has also been only partially confirmed. The analysis showed a statistically significant difference in favor of the experimental group using VR, but only in the studies using the Wong–Baker Faces Pain Rating Scale. The high pooled effect sizes in pain score, which the meta-analysis found, may suggest that VR could be more effective in distraction than other distraction methods using during medical procedures. The result of risk of bias assessment allows to believe that presented conclusions can be seen as plausible. The overall quality of the included studies was assessed as average and causes some concerns that the results are incorrect. The risk of bias assessment is so important that in the case of low-quality studies, all results and conclusions do not constitute irrefutable evidence.

An extensive systematic review by Uman et al. considered many techniques to reduce pain sensations, such as hypnosis, distraction by parents and medical staff, breathing tasks, suggestion and VR during needle-related procedures. Their conclusions suggest that distraction techniques and hypnosis should be used during needle procedures. In addition, combining distraction methods with coaching from healthcare professionals or parents was more effective than mere distraction. According to the authors, hypnosis proves to be more effective in more invasive procedures, such as a lumbar puncture. Other methods of managing pain and anxiety in these studies have proved unsuccessful [46]. Klassen et al. obtained a similar result for distraction. In their review, the effectiveness of music therapy as a distractor in medical and dental procedures was explored. An overall significant reduction in pain and anxiety was shown. Moreover, both active music therapy, with the participation of a therapist, and passive, without the intervention of the caregiver, turned out to be equally effective [47].

A study by Luo et al. [48] tried to explain the mechanism of the impact of VR on pain perception. It has been suggested that the addition of VR to analgesics represents an effective method to alleviate burn patients’ procedural pain during dressing change or physical therapy. The repeated use of VR was assessed in three of the included studies and one non-RCT and the pain reduction effect of VR remained over multiple days (up to 7 days) of testing. Likewise, a study by Hughes et al. [49] suggests that distraction-based analgesia is a form of nonpharmacological therapy that has been shown to alter the perception of acute pain by reducing the activity within pain-related brain regions. Scientists are making a lot of effort to understand how VR impacts on the perception of pain, anxiety and fear. Furthermore, a study by Hoffmann et al. [50] provides converging evidence from subjective and objective measures that VR reduces pain. In this study, the pain was an effect of thermal stimuli. Pain-related brain activity was measured for each participant during conditions of non-VR for 3.5 min and during VR for 3.5 min while in the fMRI scanning. In the non-VR condition, brain activation was found in all five brain regions of interest: the ACC, SS1, SS2, insula and thalamus. As predicted, for the group contrast comparing non-VR vs. VR, all five brain regions of interest showed statistically significant reductions in pain-related brain activity.

According to Buhle et al. the distraction phenomenon has a different mechanism of action than placebo. The justification for such a dependence is based on two characteristic phenomena. The first is the cumulative analgesic effect of distraction and placebo. The authors, based on the results of their research, indicate that placebo and distraction provide two separate routes to pain relief. In addition to summarizing the analgesic effect, the authors also performed an fMRI analysis which suggested that distraction effectively inhibits pain processing in the brain, while placebo may not significantly affect pain processing. Although both distraction and placebo lowered pain ratings, only distraction reduced the neural signature of pain by the brain [51].

Although our results suggest a statistically significant, favorable change for pain score in children, there could be some factors that may influence the results. The questionnaires and scales that are often used and the standardized research tools are subjective in nature, or may differ in the interpretation of individual participants. However, it is undisputed that the patient’s feelings and mental state in this type of research are the most important issue, so the very fact of obtaining more favorable results after a procedure using VR interventions as a distractor confirms the legitimacy of using this technology in medicine. It should be emphasized that this study was limited by the significant heterogeneity observed across studies that could not be explained by subgroup analyses or meta-regressions. This may be explained by a scarcity of literature evaluating the implementation of VR during medical procedures. The results, however, suggest that research in this approach should be expanded and VR should be introduced into treatments with young patients in order to improve the psychological comfort of children in medical facilities. The final interpretation of the test results also depends on the ES. Therefore, we also decided to calculate the ES of the included studies. The small ES in the most extensive study do not allow us to draw conclusions that clearly indicate the effectiveness of VR as a distractor.

The results of this review suggest that the pain-reducing effect of VR seems to present itself as encouraging. Although the problem of painful needle-related procedures in children is common and important, the topic is relatively poorly researched. Single studies indicate that VR also has the potential to alleviate the stress and anxiety associated with such treatments, but we cannot provide clear and strong recommendations or generalizations. This systematic review highlights the need for further research in this area. It is important to indicate which needle-related medical procedures are the most painful and stressful for young patients. Subsequently, it will be possible to create protocols using VR that will improve the children’s psychological comfort. Such an intervention could be used during procedures when children can see needles in use (e.g., blood collection, injections, vaccinations). Perhaps long-term infusions (e.g., chemotherapy) may be a good indication for the use of VR as a distractor in children. Unfortunately, the high heterogeneity of results and small sample size are a limitation for assessing the viability of VR in this type of phobia. 

However, it is worth emphasizing that VR is not the only method of distraction. A study by Aydin et al. compared three different distraction methods during venipuncture in children. The used distraction techniques were ball squeezing, balloon inflating and distraction cards. The results showed no significant difference in the children’s anxiety and pain levels [52]. Birnie et al. [53] compared other methods of distraction such as watching TV, listening to music, reading books, hypnosis or parental distraction. The results of this meta-analysis showed strong support for distraction and hypnosis for reducing pain and distress from needle procedures. However, the quality of available evidence was low. 

The results of this review may also be relevant during the ongoing COVID-19 pandemic. The majority of countries worldwide rely on available vaccines as the main approach to combating the virus. As vaccination of children and adolescents begins, VR may facilitate easier vaccine delivery to the youngest patients, as the vaccine also requires injections similar to those described in the systematic review. It is worth considering the implementation of VR in the process of mass vaccination, as the latest scientific reports suggest a significant deterioration of the mental condition of the population, in particular of pupils up to 18 years of age, and unpleasant memories of vaccination could exacerbate this [54,55].

## 5. Conclusions

Distraction has the potential to be an effective and important tool in treatments with children. VR immerses the patient, and as a result, such a distractor is an effective method in reducing pain associated with needle-related procedures in children. Four of six of the included studies noted a large effect size of this kind of intervention. Thus, VR could increase psychophysical comfort especially in young patients. However, this systematic review and meta-analysis highlight the need for more research into the use of virtual reality as a distraction. Studies on larger groups, using similar conditions, can provide unequivocal evidence of the effectiveness of VR and enable the inclusion of such intervention in standard medical procedures.

## Data Availability

Not applicable.

## Supplementary Materials

The following are available online at https://www.mdpi.com/article/10.3390/jcm10153248/s1, File S1: search strategy; File S2: the risk of bias assessment; File S3: characteristic of the included studies.
