# Supplementary material for: Virtual Reality Interventions for Needle-Related Procedural Pain, Fear and Anxiety—A Systematic Review and Meta-Analysis
Source: J Clin Med. 2021 Jul 23;10(15):3248. doi: 10.3390/jcm10153248 (PMC8347054; doi:10.3390/jcm10153248)
Supplement: Supplementary file 1 [file jcm-10-03248-s001.zip › File S1.pdf]

PUBMED

#1 “fear of needles” OR "trypanophobia” OR “needle phobia”

#2 “VR” OR “virtual therapy” OR “virtual environment” OR “virtual treatment” OR “immersive” OR “non-immersive” OR “hmd” OR “head mounted display” OR “HTC” OR “Oculus” OR “google” OR “Virtual Reality Exposure Therapy”[MeSH] OR “Smart Glasses”[MeSH] OR “Virtual Reality”[MeSH]

#3 “pain score” OR “pain level” OR “Pain”[MeSH] OR “Acute Pain”[MeSH] OR “fears” OR “fear score” OR “fear level” OR “Fear”[MeSH] OR “anxiety level” OR “anxiety score” OR “Anxiety”[MeSH] OR “pulse” OR “pulse rate” OR “Heart Rate”[MeSH] OR “Blood Pressure”[MeSH]

#4 (#1 AND #2 AND #3)

Cochrane

#1 fear of needles

#2 trypanophobia

#3 needle phobia

#4 (#1 OR #2 OR #3)

#5 MeSH descriptor: [Virtual Reality Exposure Therapy] explode all trees

#6 MeSH descriptor: [Smart Glasses] explode all trees

#7 MeSH descriptor: [Virtual Reality] explode all trees

#8 VR

#9 virtual therapy

#10 virtual environment

#11 virtual treatment

#12 immersive

#13 non-immersive

#14 hmd

#15 head mounted display

#16 HTC

#17 Oculus

#18google

#**19** {OR #5-#19}

#**20** MeSH descriptor: [Acute Pain] explode all trees

#**21** MeSH descriptor: [Pain] explode all trees

#**22** MeSH descriptor: [Fear] explode all trees

#**23** MeSH descriptor: [Anxiety] explode all trees

#**24** MeSH descriptor: [Heart Rate] explode all trees

#**25** MeSH descriptor: [Blood Pressure] explode all trees

#**26** pain score

#**27** pain level

#**28** fears

#**29** fear score

#**30** fear level

#**31** anxiety level

#**32** anxiety score

#**33** pulse

#**34** pulse rate

#**35** {OR #20-#34}

#**36** (#4 AND #19 AND #35)

Web of Science

#**1** WC= Medicine, Research & Experimental

#**2** WC= Dentistry, Oral Surgery & Medicine

#**3** WC=Medicine, General & Internal

#**4** (#1 OR #2 OR #3)

#**5** TS=("fear of needles" OR "trypanophobia" OR "needle phobia")

#**6** TS=(VR OR "virtual therapy" OR "virtual environment" OR "virtual treatment" OR immersive OR non-immersive OR hmd OR "head mounted display" OR HTC OR Oculus OR google OR "Virtual Reality Exposure Therapy" OR "Smart Glasses" OR "Virtual Reality")

#7 TS=("pain score" OR "pain level" OR Pain OR "Acute Pain" OR fears OR "fear score" OR "fear level" OR Fear OR "anxiety level" OR "anxiety score" OR Anxiety OR pulse OR "pulse rate" OR "Heart Rate" OR "Blood Pressure")

#8 (#4 AND #5 AND #6 AND #7)

Scopus

#1 "fear of needles" OR "trypanophobia" OR "needle phobia"

#2 VR OR "virtual therapy" OR "virtual environment" OR "virtual treatment" OR immersive OR non-immersive OR hmd OR "head mounted display" OR HTC OR Oculus OR google OR "Virtual Reality Exposure Therapy" OR "Smart Glasses" OR "Virtual Reality"

#3 "pain score" OR "pain level" OR Pain OR "Acute Pain" OR fears OR "fear score" OR "fear level" OR Fear OR "anxiety level" OR "anxiety score" OR Anxiety OR pulse OR "pulse rate" OR "Heart Rate" OR "Blood Pressure"

#4 (#1 AND #2 AND #3)

Embase

#1 "fear of needles" OR "trypanophobia" OR "needle phobia"

#2 'Virtual Reality Exposure Therapy'/de OR 'Smart Glasses'/de OR 'Virtual Reality'/de

#3 VR OR "virtual therapy" OR "virtual environment" OR "virtual treatment" OR immersive OR non-immersive OR hmd OR "head mounted display" OR HTC OR Oculus OR google

#4 (#2 OR #3)

#5 'Blood Pressure'/de OR 'Heart Rate'/de OR 'anxiety'/de OR 'fear'/de OR 'Acute Pain'/de OR 'Pain'/de

#6 "pain score" OR "pain level" OR fears OR "fear level" OR "fear score" OR "anxiety level" OR "anxiety score" OR pulse OR "pulse rate"

#7 (#5 OR #6)

#8 (#1 AND #4 AND #7)
