# Supplementary material for: Virtual Reality Interventions for Needle-Related Procedural Pain, Fear and Anxiety—A Systematic Review and Meta-Analysis
Source: J Clin Med. 2021 Jul 23;10(15):3248. doi: 10.3390/jcm10153248 (PMC8347054; doi:10.3390/jcm10153248)
Supplement: Supplementary file 1 [file jcm-10-03248-s001.zip › File S2.pdf]

ASSESSMENT OF RISK OF BIAS OF THE INCLUDED STUDIES

1. Caruso et al. 2019

Bias	Authors' judgement	Support for judgement
Random sequence generation (selection bias)	Low risk	Random number generator
Allocation concealment (selection bias)	Low risk	Allocation generator
Blinding of outcome assessment (detection bias)	Unclear risk	No information about blinding assessor.
Incomplete outcome data (attrition bias)	High risk	31 participants were excluded from analysis due to missing primary outcome data.
Selective reporting (reporting bias)	Low risk	The study protocol is not available but it is clear that the published reports include all expected outcomes.

2. Dumulin et al. 2019

Bias	Authors' judgement	Support for judgement
Random sequence generation (selection bias)	Low risk	Random numbers table
Allocation concealment (selection bias)	Unclear risk	No information
Blinding of outcome assessment (detection bias)	Unclear risk	No information about blinding assessor.
Incomplete outcome data (attrition bias)	Low risk	No missing outcome data
Selective reporting (reporting bias)	Low risk	The study protocol is not available but it is clear that the published reports include all expected outcomes.

3. Gerçeker et al. 2020

Bias	Authors' judgement	Support for judgement
Random sequence generation (selection bias)	Low risk	Computer generated number table
Allocation concealment (selection bias)	Low risk	Allocation generator
Blinding of outcome assessment (detection bias)	Unclear risk	The blindig of outcome assessor is unclear described
Incomplete outcome data (attrition bias)	Low risk	5 participants were excluded from analysis due to removing vr glasses during intervention.
Selective reporting (reporting bias)	Low risk	The study protocol is available. The published reports include all expected outcomes

4. Gerçeker et al. 2021

Bias	Authors' judgement	Support for judgement
Random sequence generation (selection bias)	Low risk	Randomization was performed with a computerized random–number generator
Allocation concealment (selection bias)	High risk	The allocation was based on gender and group age
Blinding of outcome assessment (detection bias)	Low risk	Data analyst was blinded
Incomplete outcome data (attrition bias)	Low risk	3 participants were excluded from analysis due to the port needle insertion procedure failure
Selective reporting (reporting bias)	Low risk	The study protocol is available. The published reports inclu de all expected outcomes

5. Gold et al. 2006

Bias	Authors' judgement	Support for judgement
Random sequence generation (selection bias)	Unclear risk	No information about randomization tool.
Allocation concealment (selection bias)	Unclear risk	No information about allocation tool.
Blinding of outcome assessment (detection bias)	Unclear risk	No information.
Incomplete outcome data (attrition bias)	Low risk	No missing outcomes.
Selective reporting (reporting bias)	Low risk	All of the study's pre–specified primary outcomes have been reported

6. Semerci et al. 2020

Bias	Authors' judgement	Support for judgement
Random sequence generation (selection bias)	Low risk	blocked randomization
Allocation concealment (selection bias)	Low risk	The allocation concealment through a computer–generated number table
Blinding of outcome assessment (detection bias)	High risk	The study was not blind;
Incomplete outcome data (attrition bias)	Low risk	1 participant from control group was excluded from analysis due to incomplete data<br>2 participants from VR group was excluded from analysis due to removing VR glasses
Selective reporting (reporting bias)	Low risk	The study protocol is available. The published reports include all expected outcomes

7. Wint et al. 2002

Bias	Authors' judgement	Support for judgement
Random sequence generation (selection bias)	Unclear risk	No information
Allocation concealment (selection bias)	Unclear risk	No information
Blinding of outcome assessment (detection bias)	Unclear risk	No information about blinding assessor
Incomplete outcome data (attrition bias)	Low risk	No missing outcome data
Selective reporting (reporting bias)	Low risk	All of the study's pre-specified primary outcomes have been reported
