# Supplementary material for: Virtual Reality Interventions for Needle-Related Procedural Pain, Fear and Anxiety—A Systematic Review and Meta-Analysis
Source: J Clin Med. 2021 Jul 23;10(15):3248. doi: 10.3390/jcm10153248 (PMC8347054; doi:10.3390/jcm10153248)
Supplement: Supplementary file 1 [file jcm-10-03248-s001.zip › File S3.pdf]

CHARACTERISTIC OF THE INCLUDED STUDIES

STUDY ID	STUDY DESIGN	PARTICIPANT	VR ENVIRONMENT	INTERVENTION	OUTCOMES	CONCLUSION
Wint et al. 2002	Pilot Study with randomization	Patients aged 10-19 with different tumors undergoing frequently Lumbar Punctures (LPs).<br><br>1. Experimental group n=17<br><br>2. Control Group n=13	immersive VR	1. Experimental Group- Virtual reality care + standard care<br>- watching a 3D video with stereo sound (“Escape”)<br>- scenarios: skiing down the Swiss Alps, explosive drag racing, a stroll down Paris sidewalks, and visions of quiet mountain streams<br><br>2. Control Group- standard care<br>- weight-based conscious sedation using fentanyl and midazolam<br>- 2.5 grams of EMLA cream applied at the spinal injection site<br>- a full explanation of the LP given to the patient and parent<br>- parental presence at the patient’s side for support	1. Primary outcomes:<br>- pain score- VAS scale (0-100)<br>- subject’s sedation level- Sedation Assessment Scale<br>- experience during the LP- the 10- item open-ended question VR questionnaire	Visual analogue scale pain scores tended to be lower in the VR group. Virtual reality glasses are a feasible, age-appropriate, nonpharmacologic adjunct to conventional care in managing pain associated with LPs in adolescents with cancer.
Caruso et al. 2020	RCT	Patients aged 7-18 undergoing vary settings vascular access in the hospital's preoperative center, interventional radiology suite, cancer center, short stay unit, and emergency department.<br><br>1. Experimental Group n=132	immersive VR	1. Experimental Group: Virtual reality care<br>- the Samsung GearVR headset<br>- one of three VR experiences: Ocean Rift, Pebbles the Penguin or Space Pups<br>- The VR intervention was carried out during the vascular access<br><br>2. Control Group: Standard Care<br>- nonstandarized, nonpharmacological standard-of-care adjuncts: nonprocedural talk, movies, CCLS, and parental presence	1. Primary outcomes:<br>- pain score- Faces Pain Scale-Revised (FPS-R)<br><br>2. Secondary outcomes:<br>- fear- Child Fear Scale (CFS)<br>- procedural compliance- modified Induction Compliance Checklist (mICC)<br>- satisfaction- satisfaction surveys<br>- incidence of adverse events	Virtual reality during vascular access in varied clinical settings did not reduce pain perception or fear but was associated with high satisfaction

		2. Control Group<br>n=127				
Dumoulin et al. 2019	RCT	Patients aged 8-17 undergoing blood work or intravenous placement in the hospital's Emergency Department.<br><br>1. Experimental group I, n=20<br><br>2. Experimental Group II, n=24<br><br>3. Experimental Group III, n=15	immersive VR	1. Experimental Group I- Virtual reality care<br>- the eMagin z800 HMD<br>- an immersive fly shooting game<br><br>2. Experimental Group II- TV minimal distraction condition<br>- watching one of two videos: Looney Tunes or Animal Planet's Funniest Animals on a portable DVD player<br><br>3. Experimental Group III- standard care<br>- the Child Life program administered by licensed Child Life specialist<br>- nonpharmacological distraction srtrategies: I-Spy Books, nonprocedural talk or 20 questions ball	1. Primary outcomes:<br>- pain score- VAS scale (0-100)<br>- anxiety score- VAS scale (0-100)<br><br>2. Secondary outcomes:<br>- patients' satisfaction- satisfaction questionnaire<br>- negative side effects- 0-10 scale	The results demonstrate the potential benefits of VR in pediatric EDs and suggest its ability to reduce aversion to painful or fearful needle-related procedures.
Gold et al. 2006	RCT	Patients from aged 8-12 undergoing intravenous (IV) placement in the hospital's Department of Radiology.<br><br>1. Experimental Group, n=10<br><br>2. Control Group, n=10	immersive VR	1. Experimental Group- standard care + VR<br>- the 5DT HMD 800<br>- the immersive "Street Luge" game<br>- the intervention began 5 minutes before the IV placement and ended 5 minutes after the procedure<br><br>2. Control Group- standard care<br>- an anesthesia spray before the IV placement<br>- the patients were given the opportunity to play with the VR for 3 min following the completion of their IV placement	1. Primary outcomes:<br>- pre-existing pain<br>- IV pain intensity<br>- past IV pain intensity<br>- affective pain intensity<br>Measured using the Wong-Baker FACES Pain Rating Scale and the Faces Pain Scale–Revised<br><br>- anxiety sensitivity- the Childhood Anxiety Sensitivity Index<br><br>- sickness feel as a result of the intervention- the Child Simulator Sickness Questionnaire	This study has demonstrated both the feasibility and utility of VR pain distraction for IV Placement in an outpatient radiology department.

					- child's engagement with the intervention- the Child Presence Questionnaire<br><br>- satisfaction- Likert-format surveys assessing behavioral distress reduction and overall satisfaction for the child, parent and nurse	
Gerçeker et al. 2021	RCT	Patients aged 6-17 undergoing Huber needle insertion into a subcutaneously implanted intravenous port from the hospital's Department of Radiology.<br><br>1. Experimental Group, n=21<br><br>2. Control Group, n=21	immersive VR	1. Experimental Group- standard care + VR<br>- Samsung Gear Oculus headset<br>- Three VR applications were used in this study; swimming with marine animals underwater (Ocean Rift), riding a rollercoaster (Rilix VR), and exploring the forest through the eyes of woodland species (In the eyes of animal).<br>- the intervention began 2/3 minutes before the IV placement and ended when procedure has been completed<br><br>2. Control Group- standard care<br>- patients and their parents were informed by the staff at least 1 h before the procedure,<br>- none pharmacological methods were used at the accessing the venous port with Huber needle	1. Primary outcomes:<br>- patient-reported pain scores- the Wong-Baker FACES (WBS) Pain Rating Scale.<br><br>2. Secondary outcomes:<br>- anxiety score- The Children's Anxiety Meter-State (CAM-S)<br>- fear score- The Child Fear Scale (CFS)	This study conducted in the Pediatric Hematology-Oncology population reveals the usability of VR distraction.
Gerçeker et al. 2020	RCT	Patients from the hospital's Blood Draw Unit aged 5-12 undergoing blood draw.<br><br>1. Experimental	immersive VR	1. Experimental Group- VR<br>- Samsung Gear Oculus headset<br>- VR-Rollercoaster<br><br>2. Experimental Group- VR<br>- Samsung Gear Oculus headset<br>- VR-Ocean Rift	1. Primary outcomes:<br>- patient-reported pain scores<br>- parent-reported pain scores<br><br>Measured using the Wong-Baker FACES (WBS) Pain	The use of VR for children receiving blood draw is an effective nonpharmacologica l method to decrease pain, fear

		Group I, n=45<br><br>2. Experimental Group II, n=45<br><br>3. Control Group, n=46		3. Control Group- standard care	Rating Scale.<br><br>- patient-reported anxiety score<br>- parent-reported anxiety score<br><br>Measured using the Children's Anxiety Meter.<br><br>- patient-reported fear score<br>- parent-reported fear score<br><br>Measured using the Child Fear Scale.	and anxiety.
Semerci et al. 2020	RCT	Patients from the hospital's Pediatric Oncology Unit aged 7-18 who required venous port access.<br><br>1. Experimental Group, n=35<br><br>2. Control Group, n=36	immersive VR	1. Experimental Group- VR<br>- the Piranha VR system<br>- the rollercoaster video<br><br>2. Control Group- standard care<br>- no pain-reducing intervention	1. Primary outcomes:<br>- patient-reported pain scores<br>- parent-reported pain scores<br><br>Measured using the Wong-Baker FACES (WBS) Pain Rating Scale.	VR is an effective method for decreasing the pain experienced by children with cancer during venous port access. It is recommended that VR should be combined with other nonpharmacological and pharmaco-logical methods to better manage pain.
